# Factors Associated with Atopic Dermatitis among Children Aged 6 to 14 Years in Alimosho Local Government, Lagos, Nigeria

**DOI:** 10.3390/children10050893

**Published:** 2023-05-17

**Authors:** Olubunmi A. Kayode, Charlotte M. Mokoatle, Phoka C. Rathebe, Thokozani P. Mbonane

**Affiliations:** Department of Environmental Health, Faculty of Health Sciences, University of Johannesburg, Johannesburg 2000, South Africa

**Keywords:** atopic dermatitis, children, allergic, skin conditions, environmental factors

## Abstract

There has been a rise in the prevalence of atopic dermatitis (AD) globally, especially in low-and middle-income countries such as Nigeria. The condition has been linked to genetic predisposes, living conditions, and environmental factors. Environmental factors are considered a significant contributor to AD in low- and middle-income countries. This study determined the prevalence of AD in south-western Nigeria and identified risk factors in home and school environments that children aged 6 to 14 years are exposed to. A cross-sectional study was adopted, and the total sample size was 349. Four randomly selected health facilities were used for the study. A questionnaire was used to determine the risk factors in the population. Data analysis was performed using the latest version of Statistical Package for Social Science (SPSS). The prevalence of atopic dermatitis in this study is 25%. Atopic dermatitis was found to be common in females (27%). According to the univariate analysis, children who lived where trucks pass on the street almost daily had the highest cases of atopic dermatitis (28%). Children with rugs in their houses (26%) and those whose houses are surrounded by bushes (26%) had higher cases of atopic dermatitis. Children who played on school grass (26%), attended creche with rubber toys (28%), and attended school where wooden chairs (28%) and chalkboards (27%) are used had a higher number of AD. Bivariate analysis showed an association between AD with a mother’s monthly income (*p* = 0.012) and eating potatoes (*p* = 0.005), fruits (*p* = 0.040), and cereal (*p* = 0.057). In the multivariate analysis, the consumption of fruits (*p* = 0.02), potatoes (*p* < 0.001), and cereal (*p* = 0.04) were identified as risk factors associated with AD. It is envisaged that the study will serve as a basis for possible research on evidence-based and primary prevention options. Hence, we recommend health education activities to empower communities to protect themselves against environmental risk factors that are preventable.

## 1. Introduction

Atopic dermatitis (AD) is a prevalent, most common chronic skin condition in children. It is a chronic inflammatory skin condition characterized by pruritus and common in pediatric dermatology clinics [1,2,3,4]. AD has a significant impact on the quality of life among children, while indirectly affecting their families [5,6]. The physical impact of this condition on children includes irritability, excessive crying, and sleep disturbances, whereas its social impact includes stigma due to visible skin conditions [7]. This worldwide skin condition burden is treatable and preventable. Preventing the occurrence of this disease would help reduce its physical, social, and economic impact [8]. Scientific evidence further shows that a combination of genetic and environmental factors contributes towards the development of AD [9].

Several cross-sectional studies have identified the association between environmental factors and AD, but only a few factors, such as air pollution and the keeping of pets, were discovered [10,11]. A study carried out in Abuja, Nigeria also noted that AD was associated with the dry season [12]. With respect to the burden of AD, there are more environmental factors yet to be identified. A study in 2019 also identified this gap and noted that further studies are required to identify other environmental factors [13]. One of the identified gaps is that guardians and parents have little knowledge about the causes of atopic dermatitis [14]. However, it is reported that some are aware of the hereditary component of AD, but believe that other factors are the main triggers [15]. Thomsen and colleagues noted that there is a sevenfold increased risk of AD in affected identical twins unlike a threefold risk in the second twins of fraternal twins [16]. Children whose parents have allergies are more likely to develop atopic dermatitis than children of parents without allergic conditions [17,18]. Despite this clear genetic predisposition for AD, evidence suggests that environmental factors contribute significantly to the development of the disease [19].

The identification of the factors triggering this disease in the home and school environments, as well as educating parents and guardians, will help them take caution while caring for their children during infancy and childhood [20]. Direct medical costs, direct family care costs, and indirect costs associated with the loss of productivity of parents may also be reduced [21].

The diagnosis of atopic dermatitis is made using the Hanifin and Rajka criteria. This requires that an individual presents with a pruritic rash and three or more of the following features: history of rash in the skin creases (fold of the elbow, behind the knees, front of the ankles, and around the neck), a personal or family history of asthma and hay fever, history of generalized dry skin (xerosis), onset before the age of 2 years, and visible flexural dermatitis [22]. In 2004, Nnoruka noted that the symptoms these children present with vary according to ethnicity [23]. However, the Hanifin and Rajka criteria remain internationally acceptable and were used in making a diagnosis of AD in this study.

Atopic dermatitis has been proven to be a dermatological disease of public health importance due to its rising prevalence over the years. The literature review showed that despite genetic factors being associated with atopic dermatitis, there seems to be environmental factors that trigger the onset of the disease. To our knowledge, there are limited studies that have been conducted in Nigeria, especially in the southwestern part, to identify these factors, hence the need for this study which determined the prevalence and identified home-related and school-related risk factors of atopic dermatitis. It also determined the association between these factors and atopic dermatitis. The aim of this study was to determine environmental factors associated with atopic dermatitis among children aged 6 to 14 years in Alimosho Local Government, Lagos, Nigeria. The specific objectives of this study were to:

(i) to determine the prevalence of AD among children aged 6 to 14 years;
(ii) to determine household risk factors of AD among study participants;
(iii) to determine school-related risk factors of AD among study participants; and
(iv) to establish the association between AD and risk factors in the study population.

## 2. Materials and Methods

### 2.1. Study Design

An observational cross-sectional study design was adopted for this research project. This study was conducted over a period of 3 months from January to March 2023.

### 2.2. Study Population

This study was conducted at four private health facilities in Alimosho Local Government of Lagos State, Nigeria. Alimosho is a Local Government Area in the Ikeja Division of Lagos State, Nigeria. It is the largest local government in Lagos, with over two million inhabitants. The study population consisted of children aged 6 to 14 years who attended the health facilities chosen for the study. The ISAAC phase-three study on atopic dermatitis was within the same age group [24]. Any children younger than 6 years or older than 14 years were excluded from this study. This study only included children aged 6 to 14 years diagnosed with atopic dermatitis and other dermatological conditions whose parents gave consent, and children whose parents did not give consent were excluded.

### 2.3. Sampling Approach

There were 12 private health facilities in Alimosho local government targeted as a study site. However, the researcher allocated numbers from one to twelve. Thereafter, three facilities were drawn by choosing every third facility on the list. The participants were purposively selected once they were diagnosed. The practicing physician gave information about the study to the caregiver of any child aged 6 to 14 diagnosed with AD and other dermatological conditions during the period of the study. The physician also identified those with atopic dermatitis by giving them a paper as ‘A’, and those who were managed for other dermatological conditions other than atopic dermatitis as ‘B’. They approached us for participation in the study after being referred by their physician. The participant selection process was repeated until the desired sample size was reached. The sample size was determined using EPI INFO 7.2 (Centre for Disease Control and Prevention). The following parameters were used when calculating the sample: the confidence level as 95%, power as 95%, prevalence in general population (unexposed) as 8.5%, prevalence in exposed as 30.4% [23]. Therefore, the total sample size calculated is 290. To compensate for non-respondent and invalid forms, the calculated sample size was increased by 20% to get the final sample size for the study, which is 349.

### 2.4. Data Collection

Interviews were conducted to collect data using a structured questionnaire with closed-ended questions. The interviews were administered by trained research assistants. The questionnaire was designed and based on the ISAAC template. The questionnaire was used to collect participants’ sociodemographic information, atopic dermatitis, school, and household environmental factors. Participants were asked about age, gender, position in the family, level of education of the parent, parent occupation, and parent income range. The atopic dermatitis criteria included any history of itchy rashes, the first age a rash was ever noticed, any history of rash in the skin creases (such as the fold of the elbow, behind the knees, front of ankles, and around the knee), any personal history of asthma, any family history of asthma, and any history of dry skin. The following household environmental related factors were collected: history of smoking in parents/guardians, history of having pets such as cats and dogs, use of rugs, fuel used for cooking, and the number of times some food items are eaten. Lastly, we collected the following school environmental factors: presence of rubber toys in creche, playing on school grass, and the use of wooden chairs and chalkboards in school.

### 2.5. Data Analysis

The variables analyzed include sociodemographic factors, factors in the home, and factors in the school environment. Cross tabulations were used to determine the prevalence and identify risk factors in home and school environments. These data were analyzed using SPSS version 28. Specific data analysis by objectives was conducted as follows:

Objective i: frequency distribution was used to quantify the prevalence of atopic dermatitis among children aged 6 to 14 years in Alimosho Local Government, Lagos, Nigeria. Tables and graphs were used to summarize the data.

Objective ii: frequency distributions, cross tabulations, and binary and multivariate logistic regression models were used to explore the relationship between atopic dermatitis among children aged 6 to 14 years in Alimosho Local Government, Lagos Nigeria, and socio-demographic factors. *p*-values (0.05) were used to test if the relationship is statistically significant.

Objective iii: frequency distributions, cross tabulations, and binary and multivariate logistic regression models were used to explore the relationship between atopic dermatitis among children aged 6 to 14 years in Alimosho Local Government, Lagos Nigeria, and risk factors in the home environment. *p*-values (0.05) were used to test if the relationship is statistically significant.

Objective iv: frequency distributions, cross tabulations, and bivariate and multivariate logistic regression models were used to explore the relationship between atopic dermatitis among children aged 6 to 14 years in Alimosho Local Government, Lagos Nigeria, and risk factors in the school environment. *p*-values (0.05) were used to test if the relationship is statistically significant.

### 2.6. Ethical Consideration

The study obtained ethical clearance from the University of Johannesburg, Faculty of Health Sciences, Research Ethics Committee (REC-1684-2022, 23 August 2022). The Nigerian Institution of Medical Research, Institutional Review Board (IRB/22/052, 24 November 2022) gave permission for the researchers to conduct the study and approach the participants. The parents or caregivers of the children gave consent to participate in this study.

## 3. Results

These results are presented according to the objectives of the study, and these include: to determine the prevalence, household risk factors, and school-related risk factors of AD among children aged 6 to 14 years in Alimosho Local Government, Lagos, Nigeria. Lastly, it presents the association between AD and risk factors in the study population. There were 86 (25%) participants that had AD, whereas there were 263 (75%) participants without AD. Therefore, the prevalence of AD in this study was 25%.

### 3.1. Participants’ Characteristics

As presented in the Table 1 below, the age group 9–11 years had the highest number of cases of atopic dermatitis, which was 27% (*n* = 31). More cases (28% (*n* = 44)) of AD occurred in females, unlike in males with 22% (*n* = 42) of cases. The statistical comparison between the characteristics of study participants was not significant; however, the income of mothers representing cases was significantly different to the referent group (*p* < 0.048).

### 3.2. Household-Related Risk Factors

Children whose parents have no specific fuel for cooking have the highest number of cases, at 27% (*n* = 27). Additionally, children who live where trucks pass on their street almost every day showed the highest number of cases, at 28% (*n* = 26). Those children who do not have cats and dogs in their homes have the highest number of cases, at 27% (*n* = 44) and 26% (*n* = 42), respectively. Those whose mothers/female guardians do not smoke have the highest number of cases, at 29% (*n* = 51), whereas there was no difference in percentage between children of fathers/male guardians who smoke (25% (*n* = 45)). Children who live in houses with bushes around have a higher percentage, at 26% (*n* = 44). Children who do not use cleaning agents such as JIK in their homes have a higher percentage, at 25% (*n* = 42). Children who did not have rubber toys in their house have a higher number of AD cases, at 25% (*n* = 43). Children who use rugs in their houses have a higher number of AD cases, at 26% (*n* = 48). Children who ate seafood and nuts about 4–6 times a week had the highest percentage, at 33% (*n* = 30) and 27% (*n* = 22), respectively. Children who ate meat, vegetables, butter, and eggs more than six times in week have the highest percentage, at 29% (*n* = 25), 26% (*n* = 27), 25% (*n* = 22), and 28% (*n* = 26), respectively. Children who ate cereal and milk about 1–3 times in a week also have the highest percentage of AD cases, at 34% (*n* = 29) and 29% (*n* = 28). Children who did not eat fruit, pasta, rice, butter, margarine, and potatoes have the highest percentage, at 33% (*n* = 32), 28% (*n* = 26), 28% (*n* = 26), 25% (24), 30% (27), and 34% (*n* = 32), respectively. Appendix A (Table A1) gives a detailed description of household-related risk factors.

### 3.3. School-Related Risk Factors

From Table 2 below, it can be deduced that children who played on school grass, attended creche with rubber toys, and attended schools where wooden chairs and chalkboards were used have a higher number of AD cases, at 26% (*n* = 40), 26% (*n* = 51), 28% (*n* = 52), and 28% (*n* = 48), respectively. On the other hand, children who did not attend school surrounded by bushes and who did not attend school surrounded by stagnant water also have a higher percentage of AD cases, at 26% (*n* = 45) and 28% (*n* = 51), respectively.

### 3.4. Association between Atopic Dermatitis and Risk Factors

From Table 3 below, it can be inferred from the multivariate analysis that the consumption of fruit (1–3 times a week) (*p* = 0.02), potatoes (4–6 times a week) (*p* < 0.001), and cereal (1–3 times a week) (*p* = 0.04) were associated with AD in this study.

## 4. Discussion

This study’s objectives were to determine the prevalence of atopic dermatitis and identify home- and school-related risk factors that predispose children in Alimosho Local Government, Lagos, Nigeria to atopic dermatitis and the association of these risk factors to the disease under study. A total of 349 participants were included in the study: 86 participants had atopic dermatitis, whereas 263 participants did not have atopic dermatitis. The prevalence of atopic dermatitis in this study was 25%. This finding is higher than other studies conducted in Nigeria, which reported a prevalence of 13% and 9% in Northern and Southern Nigeria, respectively [25]. However, it was lower than in a South African study that reported 60.1% prevalence [26]. This study was conducted during the dry season and might have brought about the increased prevalence noted in this study. A study in Abuja, Nigeria noted that there is an increased risk of developing AD during the dry season [12] because of the irritation to the skin caused by dry conditions.

This study shows that atopic dermatitis was common among females (27.5%) compared to males (22.2%), with a ratio of 0.8 (male) to 1 (female). This study’s findings were similar to a local study conducted elsewhere in Nigeria among children aged 0–16 years that showed a preponderance ratio to females [24], whereas another local study conducted in northern Nigeria showed that AD was common in males compared to females [27]. This might be due to the cultural difference that permits males to move freely unlike females in the northern part of the country. Therefore, males could report to hospitals more than females.

Children who live where trucks pass almost every day on the street had a higher number of AD cases (*n* = 26; 28%). Previous studies have linked frequent truck movement to air pollution on untarred roads in residential areas [28]. Consequently, air pollution is a known risk factor for AD [29]. They also argued that air pollutants appear to be one of the risk factors for developing AD. Furthermore, air pollutant exposure can lead to an imbalance between oxidants and antioxidants, thereby culminating in oxidative stress on the skin that presents as feature of AD [30]. Children who live in houses surrounded by bushes also had a higher percentage of AD (26%) (*n* = 44). Elsewhere, Montealegre and colleagues noted that patients with AD showed positive skin reactions to animal, plant, and fungal allergens [31]. Exposure to these allergens due to environmental surroundings could contribute to triggering allergic reactions that result in exacerbating AD [31].

Children who played on school grass, attended creche with rubber toys, and attended schools with wooden chairs and chalkboards had a higher prevalence, at 26.3%, 26.3%, 27.7%, and 26.7%, respectively. Werner and colleagues argued that grass pollen is one of the factors that predisposes an individual to develop AD, as his study revealed a worsening of AD when participants were exposed to grass pollen [32], and also noted that rubber materials such as rubber toys contain latex [33]. A Bolivian case study reported that wood can cause allergies, as it contains allergens such as quinones, stilbenes, phenols, and terpenes [34]. They reported that the allergens found in the wood led to the case developing of chronic dermatitis [34]. Lastly, metals in blackboard chalk are likely to trigger AD due to allergic reactions [35].

The mother’s employment status (*p* = 0.038) was found to be associated with AD. In addition, the mother’s monthly income (AOR = 3.66, 95%CI:1.67, 7.80) was associated with AD, as children of mothers who are paid between 50,000 naira and 100,000 naira are 3.66 times at risk of developing AD. This shows that AD is associated with higher maternal socio-economic status [32]. Similar to the findings from a study conducted in the city of Tehran among children aged 6 to 14 years old, socio-economic was a risk factor for AD [36]. It might be that people with high socio-economic status are able to purchase more materials and food that expose the child to several allergens.

In this study, consuming fruit 2–4 times a week (*p* = 0.02) and potatoes 4–6 times a week (*p* < 0.001) were associated with AD. This could have been caused by poor food hygiene, where fruits and potatoes were not washed properly, leading to allergic reactions that can contribute to the development of AD. Lastly, eating cereal 1–3 times a week (*p* = 0.04) was also associated with AD in the study. In most instances, milk is used when eating cereal, and there are people reported to experience allergic reactions to milk or unpasteurized milk, which may lead to skin conditions such as AD.

## 5. Strengths and Limitations

This study was conducted at four private health facilities in Alimosho Local Government, Lagos State, and focuses on children attending these health facilities. It also concentrated on one state in Nigeria. Therefore, the results cannot be generalized to other states and countries. If this study was conducted in public hospitals and rural areas, more predisposing factors might have been identified. Children spend most of their time at home and school; hence, this study identified risk factors in the home and school environment that predispose children to develop atopic dermatitis. Parents would be informed of these risk factors and would be advised to reduce or prevent exposure of their children to them. School owners would be educated and enlightened on how some factors in the school environment can predispose a child to this chronic skin disease and its possible physical, social, and mental impacts on such children. Policy makers would be advised to make policies on what the school environment should look like.

## 6. Conclusions

This study has drawn attention to the factors around the home such as trucks passing in the street and bushes around the house, as they are more likely to lead to atopic dermatitis compared to those within the home environment. There is a need to encourage the public to look outward and pay attention to the surroundings where they live to reduce the incidence of atopic dermatitis. Schools also need advancement in their technology by using markers, plastic chairs, and tables, and ensuring a tidy environment. However, this study found an association between AD with the consumption of fruit, potatoes, and cereal, highlighting food hygiene, which could have led to the development of AD in this study. Health education on food safety is recommended to ensure that communities protect themselves against preventable environmental risks. Further studies need to be carried out on a larger scale and include several local governments in the state.

## Figures and Tables

**Table 1:** Characteristics of study participants.

Variable	Total *N* (%)	Atopic Dermatitis *N* (%)	No Atopic Dermatitis *N* (%)	*p*-Value
Age				
6–8 Years	131 (38%)	32 (24%)	99 (76%)	0.773
9–11 Years	116 (33%)	31 (27%)	85 (73%)	
12–14 Years	102 (29%)	23 (23%)	79 (77%)	
Gender				
Male	189 (54%)	42 (22%)	147 (78%)	0.254
Female	160 (46%)	44 (28%)	116 (72%)	
Maternal educational status				
No education	57 (16%)	14 (25%)	43 (75%)	0.172
Primary level	77 (22%)	19 (25%)	58 (75%)	
Secondary	78 (22%)	13 (17%)	65 (83%)	
Tertiary	66 (19%)	23 (35%)	43 (65%)	
Post-Tertiary	71 (20%)	17 (24%)	54 (76%)	
Paternal educational status				
No education	56 (16%)	15 (27%)	41 (73%)	0.169
Primary level	63 (18%)	9 (14%)	54 (86%)	
Secondary	83 (24%)	18 (22%)	65 (78%)	
Tertiary	72 (21%)	21 (29%)	51 (71%)	
Post-Tertiary	75 (22%)	23 (31%)	52 (69%)	
Maternal employment status				
Employed	115 (33%)	31 (27%)	84 (73%)	0.235
Self-employed	123 (35%)	37 (30%)	86 (70%)	
Not employed	111 (32%)	18 (16%)	93 (84%)	
Paternal employment status				
Employed	122 (35%)	32 (26%)	90 (74%)	0.235
Self-employed	104 (30%)	30 (29%)	74 (71%)	
Not employed	123 (35%)	24 (19%)	99 (81%)	
Mother’s monthly income				
<10,000	90 (26%)	28 (31%)	62 (69%)	0.048 *
10,000–50,000	84 (24%)	25 (30%)	59 (70%)	
>50,000–100,000	93 (27%)	14 (15%)	79 (85%)	
>100,000	82 (24%)	19 (23%)	63 (77%)	
Father’s monthly income				
<10,000	88 (25%)	17 (19%)	71 (81%)	0.485
10,000–50,000	83 (24%)	23 (28%)	60 (72%)	
>50,000–100,000	77 (22%)	22 (29%)	55 (71%)	
>100,000	101 (29%)	24 (24%)	77 (76%)	
Position of child in the family				
First	101 (29%)	27 (27%)	74 (73%)	0.837
Intermediate	124 (36%)	30 (24%)	94 (76%)	
Last	124 (36%)	29 (23%)	95 (77%)	

* Statically significance at 0.050.

**Table 2:** School-related Risk Factors.

Variable	Total *N* (%)	Atopic Dermatitis *N* (%)	No Atopic Dermatitis *N* (%)	*p*-Value
School grass				
Yes	152 (44%)	40 (26%)	112 (74%)	0.524
No	197 (56%)	46 (23%)	151 (77%)	
Surrounding bushes				
Yes	175 (50%)	41 (23%)	134 (77%)	0.598
No	174 (50%)	45 (26%)	129 (74%)	
Stagnant water				
Yes	166 (48%)	35 (21%)	131 (79%)	0.142
No	183 (52%)	51 (28%)	132 (72%)	
Rubber toys				
Yes	194 (56%)	51 (26%)	143 (74%)	0.424
No	155 (44%)	35 (23%)	120 (77%)	
Wooden chairs				
Yes	188 (54%)	52 (28%)	136 (72%)	0.157
No	161 (46%)	34 (21%)	127 (79%)	
Chalkboard				
Yes	180 (52%)	48 (27%)	132 (73%)	0.365
No	169 (48%)	38 (23%)	131 (77%)	

**Table 3:** Multivariate logistics analysis between atopic dermatitis and risk factors.

Risk Factor	Crude Odds Ratios	*p*-Value	95% Confidence Intervals		Adjusted Odds Ratios	*p*-Value	95% Confidence Intervals	
			Lower	Higher			Lower	Higher
Weekly fruit consumption								
Less than 1	Reference							
1–3	1.71	0.11	0.88	3.32	1.64	0.22	0.74	3.66
4–6	1.92	0.06	0.98	3.75	2.60	0.02 *	1.14	5.91
>6	1.91	0.06	0.97	3.78	2.05	0.07	0.94	4.48
Weekly cereal consumption								
Less than 1	Reference							
1–3	0.53	0.06	2.73	1.03	0.45	0.04 *	0.20	0.99
4–6	0.78	0.51	0.38	1.62	0.77	0.56	0.31	1.88
>6	1.14	0.72	0.56	2.30	1.26	0.58	0.55	2.88
Weekly potatoes consumption								
Less than 1	Reference							
1–3	1.27	0.45	0.68	2.38	1.23	0.59	0.58	2.59
4–6	3.62	<0.001	1.69	7.78	4.49	<0.001 *	1.88	10.73
>6	1.94	0.06	0.98	3.86	1.96	0.11	0.86	4.46

* Statistically significant (0.005).

## Data Availability

The data presented in this study can be made available on request from the corresponding author.

## Appendix A

**Table A1:** Household-related Risk Factors.

Variable	Total *N* (%)	Atopic Dermatitis *N* (%)	No Atopic Dermatitis *N* (%)	*p*-Value
Cooking fuel				
Electricity	85 (24%)	18 (21%)	67 (79%)	0.822
Gas	76 (22%)	20 (26%)	56 (74%)	
Open fires	87 (25%)	21 (24%)	66 (76%)	
Others	101 (29%)	27 (27%)	74 (73%)	
Frequency of passing trucks				
Never	81 (23%)	19 (23%)	62 (77%)	0.745
Seldom	84 (24%)	22 (26%)	62 (74%)	
Frequently	90 (26%)	19 (21%)	71 (79%)	
Almost	94 (27%)	26 (28%)	68 (72%)	
Cat				
Yes	188 (54%)	42 (22%)	146 (78%)	0.281
No	161 (46%)	44 (27%)	117 (73%)	
Dog				
Yes	190 (54%)	44 (23%)	146 (77%)	0.482
No	159 (46%)	42 (26%)	117 (74%)	
Maternal smoking				
Yes	173 (50%)	35 (20%)	138 (80%)	0.058
No	176 (50%)	51 (29%)	125 (71%)	
Paternal smoking				
Yes	182 (52%)	45 (25%)	137 (75%)	0.970
No	167 (48%)	41 (25%)	126 (75%)	
Surrounding bushes				
Yes	166 (48%)	44 (26%)	122 (74%)	0.441
No	183 (52%)	42 (23%)	141 (77%)	
Disinfectant use				
Yes	183 (52%)	44 (24%)	139 (76%)	0.785
No	166 (48%)	42 (25%)	124 (75%)	
Rubber toys				
Yes	176 (50%)	43 (24%)	133 (76%)	0.927
No	173 (50%)	43 (25%)	130 (75%)	
Rug				
Yes	182 (52%)	48 (26%)	134 (74%)	0.433
No	167 (48%)	38 (23%)	129 (77%)	
Meat meal				
0	79 (23%)	22 (28%)	57 (72%)	0.492
1–3	85 (24%)	19 (22%)	66 (78%)	
4–6	98 (28%)	20 (20%)	78 (80%)	
>6	87 (25%)	25 (29%)	62 (71%)	
Seafood meal				
0	66 (19%)	14 (21%)	52 (79%)	0.199
1–3	76 (22%)	16 (21%)	60 (79%)	
4–6	91 (28%)	30 (33%)	61 (67%)	
>6	116 (33%)	26 (22%)	90 (78%)	
Fruit meal				
0	96 (28%)	32 (33%)	64 (67%)	0.139
1–3	84 (24%)	19 (23%)	65 (77%)	
4–6	87 (25%)	18 (21%)	69 (79%)	
>6	82 (25%)	17 (21%)	65 (79%)	
Vegetables meal				
0	67 (19%)	16 (24%)	51 (76%)	0.954
1–3	93 (27%)	23 (25%)	70 (75%)	
4–6	87 (25%)	20 (23%)	67 (77%)	
>6	102 (29%)	27 (26%)	75 (74%)	
Cereal meal				
0	94 (27%)	20 (21%)	74 (79%)	0.111
1–3	86 (25%)	29 (34%)	57 (66%)	
4–6	70 (20%)	18 (26%)	52 (74%)	
>6	99 (28%)	19 (19%)	80 (81%)	
Pasta meal				
0	93 (27%)	26 (28%)	67 (72%)	0.495
1–3	84 (24%)	20 (24%)	64 (76%)	
4–6	92 (26%)	25 (27%)	67 (73%)	
>6	80 (23%)	15 (19%)	65 (81%)	
Rice meal				
0	94 (27%)	26 (28%)	68 (72%)	0.831
1–3	90 (26%)	20 (22%)	70 (78%)	
4–6	74 (21%)	17 (23%)	57 (77%)	
>6	91 (26%)	23 (25%)	68 (75%)	
Butter meal				
0	96 (28%)	24 (25%)	72 (75%)	0.998
1–3	81 (23%)	20 (25%)	61 (75%)	
4–6	84 (24%)	20 (24%)	64 (76%)	
>6	88 (25%)	22 (25%)	66 (75%)	
Margarine meal				
0	89 (26%)	27 (30%)	62 (70%)	0.307
1–3	97 (29%)	21 (22%)	76 (78%)	
4–6	76 (22%)	21 (28%)	55 (72%)	
>6	87 (25%)	17 (20%)	70 (80%)	
Nut meal				
0	71 (20%)	19 (27%)	52 (73%)	0.597
1–3	100 (29%)	26 (26%)	74 (74%)	
4–6	81 (23%)	22 (27%)	59 (73%)	
>6	97 (28%)	19 (20%)	78 (80%)	
Potatoes meal				
0	93 (27%)	32 (34%)	61 (66%)	0.005
1–3	89 (26%)	26 (29%)	63 (71%)	
4–6	87 (25%)	11 (13%)	76 (87%)	
>6	80 (23%)	17 (21%)	63 (79%)	
Milk meal				
0	81 (23%)	21 (26%)	60 (74%)	0.274
1–3	98 (28%)	28 (29%)	70 (71%)	
4–6	79 (22%)	13 (16%)	66 (84%)	
>6	91 (26%)	24 (26%)	67 (74%)	
Egg meal				
0	87 (25%)	21 (24%)	66 (76%)	0.776
1–3	82 (24%)	20 (24%)	62 (76%)	
4–6	88 (25%)	19 (22%)	69 (78%)	
>6	92 (26%)	26 (28%)	66 (72%)	
